# A comparison of health status in patients meeting alternative definitions for chronic fatigue syndrome/myalgic encephalomyelitis

**DOI:** 10.1186/1477-7525-12-64

**Published:** 2014-04-30

**Authors:** Samantha C Johnston, Ekua W Brenu, Sharni L Hardcastle, Teilah K Huth, Donald R Staines, Sonya M Marshall-Gradisnik

**Affiliations:** 1 Griffith Health Institute, School of Medical Sciences, National Centre for Neuroimmunology and Emerging Diseases, Griffith University, Parklands, QLD 4222, Australia; 2 Gold Coast Public Health Unit, Queensland Health, Robina, QLD, Australia

**Keywords:** Chronic fatigue syndrome, Disability, Myalgic encephalomyelitis, Health-related quality of life

## Abstract

**Background:**

Several diagnostic definitions are available for Chronic Fatigue Syndrome/Myalgic Encephalomyelitis (CFS/ME) that varies significantly in their symptom criteria. This pilot study was conducted to determine whether simple biological and clinical measures differed between CFS/ME patients meeting the 1994 Centres for Disease Control and Prevention (CDC) criteria, the International Consensus Criteria (ICC), as well as healthy controls.

**Methods:**

A total of 45 CFS/ME patients and 30 healthy controls from the South East Queensland region of Australia provided a blood sample, reported on their current symptoms, as well as aspects of their physical and social health using the Short-Form Health Survey (SF-36), and the World Health Organisation Disability Adjustment Schedule 2.0 (WHO DAS 2.0). Differences were examined using independent sample t-testing.

**Results:**

Patients fulfilling the ICC definition reported significantly lower scores (p < 0.05) for physical functioning, physical role, bodily pain, and social functioning than those that only fulfilled the 1994 CDC definition. ICC patients reported significantly greater (p < 0.05) disability across all domains of the WHO DAS 2.0.

**Conclusions:**

These preliminary findings suggest that the ICC identifies a distinct subgroup found within patients complying with the 1994 CDC definition, with more severe impairment to their physical and social functioning.

## Introduction

The term Chronic Fatigue Syndrome/Myalgic Encephalomyelitis (CFS/ME) first appeared in the literature in 1988 when the Centres for Disease Control and Prevention (CDC) described an illness of debilitating fatigue accompanied by a various combination of symptoms [1]. Throughout the 1950s to 1980s however, outbreaks of CFS/ME-like illness have been reported as Bornholm disease [2], Iceland disease [3], the Royal Free Hospital epidemic [4], as well as Chronic Epstein Barr Virus Syndrome [5]. In recent decades, several formal case definitions have been released for CFS/ME [6-12], and each differ significantly in the symptoms they emphasise, as well as their exclusion criteria [13]. The most common definition is the 1994 CDC, which requires the presence of debilitating fatigue of 6 months, and at least four of eight, mostly flu-like symptoms [8]. It was primarily developed for the selection of adult cases in research however, concerns have been raised on its selection of widely heterogeneous patients [14,15].

A more stringent definition, known as the Canadian Consensus Criteria was released in 2003 [7], primarily for diagnosis in clinical settings. Criteria included core symptoms found in the 1994 CDC such as debilitating fatigue for 6 months, post-exertional malaise, sleep dysfunction, and pain, as well as symptoms relating to dysfunction of the neurological, autonomic, neuroendocrine, and immune systems. Its application in research however, suggests that patients fulfilling the Canadian definition have more severe impairment to their physical functioning and cognition than 1994 CDC defined patients [16,17].

The Canadian definition was revised in 2011 and renamed the International Consensus Criteria (ICC) [6]. The most significant change in the ICC definition was the removal of criteria for fatigue, with a revised emphasis on fatigability, referred to as post-exertional neuroimmune exhaustion (PENE). Accompanying symptoms are then categorised into three pathophysiologies relating to dysfunction of the neurological system, immune and gastro-intestinal system, and deregulation of energy metabolism and ion transportation. This latest definition has yet to be applied regularly in CFS/ME research.

A major criticism of the 1994 CDC definition is that it has remained the most common criteria for CFS/ME due to consensus [18]. The ICC was proposed based on collective empirical findings on dysfunction found in CFS/ME patients fulfilling broader definitions of the illness [19-29]. These findings, however, may be more prominent in a more homogenous sample. The potential of the ICC to identify a distinct subgroup of CFS/ME may, therefore, enhance the opportunity of discovering a unique biological marker for the illness. The aim of this study was to compare patients fulfilling the 1994 CDC definition, the ICC definition, and healthy controls. It examined whether differences could be found in standard blood tests for screening of the disease that are recommended for differential diagnosis of CFS/ME [30-32]. It also examined impairments using the Short Form 36 Item Health Survey (SF-36) [33], and the World Health Organisation’s Disability Adjustment Schedule 2.0 (WHO DAS 2.0) [34]. These findings provide the first preliminary data available regarding standard biological and clinical measures in CFS/ME patients complying with the 1994 CDC and ICC definitions.

## Method

The study involved a blood sample and cross-sectional survey of participants self-reporting a current diagnosis of CFS/ME, and healthy controls, aged 18 to 64 years. Participants were part of a larger study examining immunological markers and were recruited from support networks in the South East Queensland region. Written consent was obtained from all eligible participants before collecting a blood sample to measure their full blood count (FBC), erythrocyte sedimentation rate (ESR) and electrolytes. Participants were also required to complete a hard copy of self-reporting measures. This included the National Centre for Neuroimmunology and Emerging Diseases (NCNED) CFS/ME questionnaire developed by the authors. Within the questionnaire was a checklist consisting of a total of 53 symptoms derived from the 1994 CDC [8], Canadian [7] and ICC definitions [6]. Participants first had to indicate how many months they had been experiencing chronic, debilitating fatigue. Participants then selected symptoms from the checklist that had been present and disruptive during the past 30 days. They were also asked to list their current medications and medical history including psychiatric conditions.

To be confirmed as a CFS/ME patient, current symptoms had to comply. To meet with the 1994 CDC definition, participants had to experience a debilitating fatigue for at least 6 months and four or more of the following symptoms: post-exertional malaise, difficulties with short term memory or concentration, unrefreshing sleep, sore throat, muscle pain, joint pain, headaches, and tender lymph nodes. According to the Canadian definition, patients had to experience debilitating fatigue for at least 6 months, post-exertional malaise, unrefreshed sleep, and problems with pain (muscle, joint, and/or headache). Further, patients had to indicate at least two neurocognitive symptoms, and at least one symptom from two of the remaining categories of autonomic, neuroendocrine, and immune manifestations from the checklist. To comply with the ICC definition, patients had to meet all 5 symptoms relating to PENE, neurological impairments (at least one symptom from three subcategories); immune, gastro-intestinal, and genitourinary impairments (at least one symptom from three subcategories); and energy metabolism, ion transportation impairments (one symptom).

Those reporting physical and psychiatric conditions that could potentially explain their symptoms were excluded from the study. Secondary screening of reported medications was also conducted, and patients on hormone therapy were also excluded. Healthy controls were defined as having no reported signs of illness. Participants also completed self-reporting measures of their health according to the SF-36 [33] and the WHO DAS 2.0 [34] surveys. The SF-36 has previously been examined in patients fulfilling the 1994 CDC [8] and Canadian criteria [35,36]. It investigates eight subscales according to: physical functioning, role limitations due to physical problems, bodily pain, general perception of physical health, vitality, social functioning, role imitations due to emotional problems, and general perception of mental health [33]. Scoring ranged between 0 and 100, with lower values representing greater impairment. The WHO DAS 2.0 assesses patients according to its framework for the International Classification for Functioning, Disability and Health (ICF), but has yet to be applied in CFS/ME patients [37]. It consists of six domains to assess difficulties in health relating to communication, mobility, self-care, interpersonal relations, life activities, and participation in society, over the past 30 days. Scoring also ranged between 0 and 100, with higher values indicating greater impairment.It was observed that all patients that complied with the ICC definition also fulfilled the 1994 CDC definition. Based on this, the study used three independent groups for analysis: patients conforming only to the 1994 CDC definition, patients that fulfilled the ICC definition, and healthy controls. Five cases in the 1994 CDC group were found to also comply with the Canadian definition, but not the ICC definition. Due to low statistical power of this sample alone, these cases remained in the 1994 CDC group for analysis. SPSS v.22 was used to compare mean scores on the SF-36 and WHO DAS 2.0 between 1994 CDC and ICC patient groups, and between all patients and healthy controls, using independent sample t-testing. Categorical variables were analysed using chi-squared test when appropriate. Using G*Power 3.1.9.2, a sample size of at least 18 per group, at a power of 0.8 was deemed acceptable to observe a large effect size in this pilot study. Results were considered significant at the p < 0.05 level and highly significant at the p < 0.001 level. The study was approved by the Griffith University Human Research Ethics Committee.

## Results

Of 45 self-reporting CFS/ME patients recruited into the study, 4 did not comply with any CFS/ME definitions and were excluded from analysis. Of the 41 patients included, 19 reported symptoms that only fulfilled the 1994 CDC definition, and 22 fulfilled the ICC definition. The 30 healthy controls recruited remained in the study as they indicated no signs of illness. Basic characteristics and standard blood results for each study group are presented in Table 1. The age distribution was similar between the three study groups, but a significantly higher number of females (p < 0.05) was found among ICC patients. The mean duration of illness of approximately 19 years was the same between the two patient groups. Significant differences (p < 0.05) were detected between all patients and healthy control groups in Haemoglobin, Haematocrit, and Red Cell Count with higher levels found in healthy controls. Patients also exhibited significantly higher Erythrocyte Sedimentation Rates (ESR) in comparison to healthy controls. No difference however, was found between 1994 CDC and ICC patients across these markers.

**Table 1:** Characteristics of 1994 CDC patients, ICC patients, and healthy controls

**Parameters**	**1994 CDC patients (n = 19)**	**ICC patients (n = 22)**	**Healthy controls (n = 30)**	**Sig.**
Age, mean (SD)	50.7 (7.4)	49.3 (13.2)	49.7 (10.9)	
Gender (% female)	68%	95%	66%	<0.05^a^
Illness duration (years), mean (SD)	18.9 (13.6)	19.0 (10.2)	n/a	
Haemoglobin (g/L) mean (SD)	134.9 (13.6)	132.9 (11.9)	140.7 (12.6)	<0.05^b^
White cell count (x10^9^/L) mean (SD)	6.1 (1.5)	6.1 (1.9)	6.0 (1.5)	
Platelets (×10^9^/L) mean (SD)	247.9 (66.8)	276.7 (68.5)	235.5 (48.2)	
Haematocrit mean (SD)	0.41 (0.04)	0.40 (0.03)	0.43 (0.03)	<0.05^b^
Red cell count (×10^12^/L) mean (SD)	4.45 (0.4)	4.40 (0.4)	4.65 (0.43)	<0.05^b^
Mean corpuscular volume (×10^9^/L)	91.8 (3.6)	91.6 (2.9)	92.0 (2.5)	
Neutrophils (×10^9^/L) mean (SD)	3.75 (1.3)	3.48 (1.6)	3.81 (1.28)	
Lymphocytes (×10^9^/L) mean (SD)	1.83 (0.4)	2.04 (0.8)	1.72 (0.43)	
Monocytes (×x10^9^/L) mean (SD)	0.30 (0.1)	0.34 (0.1)	0.32 (0.12)	
Eosionophils (×10^9^/L) mean (SD)	0.17 (0.1)	0.15 (0.1)	0.15 (0.11)	
Basophils (×10^9^/L) mean (SD)	0.03 (0.03)	0.03 (0.03)	0.03 (0.02)	
ESR (mm/Hr) mean (SD)	15.5 (11.2)	15.7 (13.8)	10.2 (8.4)	<0.05^b^
Sodium (mmol/L) mean (SD)	138.6 (1.3)	130.8 (30.0)	138.2 (1.8)	
Potassium (mmol/L) mean (SD)	4.0 (0.3)	9.3 (23.2)	4.0 (0.3)	
Chloride (mmol/L) mean (SD)	104.1 (2.0)	99.6 (17.5)	103.6 (2.5)	
Bicarbonate (mmol/L) mean (SD)	26.7 (2.1)	24.7 (5.3)	26.5 (2.6)	
Anions (mmol/L) mean (SD)	7.9 (1.3)	8.6 (2.5)	8.3 (1.7)	

^a^Significant difference between 1994 CDC and International patient groups.

^b^Significant difference between all patient and healthy control groups.

The results for the SF-36 are presented in Table 2. CFS/ME patients complying with either of the study criteria reported significantly lower scores for all eight SF-36 subscales (p < 0.001, except p < 0.05 for mental health), when compared to healthy controls. Among the patients, those that fulfilled the ICC definition reported significantly lower scores (p < 0.05) for physical functioning, physical role, bodily pain, and social functioning than those that only fulfilled the 1994 CDC criteria.

**Table 2:** SF-36 Scores of 1994 CDC defined patients, ICC defined patients, and healthy controls

**Scores**	**1994 CDC patients mean (SD)**	**ICC patients mean (SD)**	**Healthy controls mean (SD)**	**1994 CDC vs ICC**	**1994 CDC vs controls**	**ICC vs controls**
Physical functioning	58.7 (20.9)	35.0 (23.3)	96.1 (8.4)	p = 0.002	p = 0.000	p = 0.000
Physical role	21.1 (29.2)	1.25 (5.6)	96.4 (14.8)	p = 0.005	p = 0.000	p = 0.000
Bodily pain	62.9 (24.6)	44.8 (26.2)	94.3 (9.6)	P = 0.030	p = 0.000	p = 0.000
General health	36.8 (21.3)	31.3 (21.5)	82.5 (9.6)	p > 0.05	p = 0.000	p = 0.000
Vitality	26.6 (15.4)	19.2 (18.0)	67.6 (15.9)	p > 0.05	p = 0.000	p = 0.000
Emotional role	52.6 (46.2)	47.4 (48.8)	92.0 (21.2)	p > 0.05	p = 0.000	p = 0.000
Social functioning	26.8 (21.4)	19.2 (15.6)	94.9 (10.6)	p = 0.002	p = 0.000	p = 0.000
Mental health	66.2 (21.2)	62.5 (22.9)	79.1 (14.6)	p > 0.05	p = 0.018	p = 0.003

Lower scores indicate greater impairment.

Table 3 presents the results of the WHO DAS 2.0. The 1994 CDC patients reported significantly higher scores (p < 0.05) for all disability domains compared to healthy controls, except for self-care. ICC patients however, differed significantly across all domains. Significant differences were also found in domains of cognition, mobility, self-care (p < 0.001), as well as life activities, and participation (p < 0.05) between 1994 CDC and ICC patients.

**Table 3:** WHO DAS 2.0 scores in 1994 CDC patients, ICC patients, and healthy controls

**Scores**	**1994 CDC patients mean (SD)**	**ICC patients mean (SD)**	**Controls mean (SD)**	**1994 CDC vs ICC**	**1994 CDC vs controls**	**ICC vs controls**
Cognition	22.6 (16.2)	43.5 (17.6)	4.0 (5.6)	p = .000	p = 0.000	p = 0.000
Mobility	27.1 (17.6)	48.2 (15.5)	1.4 (3.0)	p = .000	p = 0.000	p = 0.000
Self-care	14.0 (9.6)	22.2 (16.5)	16.5 (0.7)	p = .000	p > 0.05	p = 0.000
Getting along	15.6 (15.2)	44.2 (22.7)	22.7 (5.5)	p = .000	p = 0.007	p = 0.000
Life activities	39.9 (25.9)	63.1 (23.8)	23.8 (6.3)	p = .010	p = 0.000	p = 0.000
Participation	38.2 (20.4)	53.9 (16.5)	16.5 (3.6)	p = .011	p = 0.000	p = 0.000

Higher scores indicate greater impairment.

## Discussion

This is the first study to examine CFS/ME patients that fulfil the ICC definition in an Australian sample. As all ICC defined patients complied with 1994 CDC definition, this study supports findings that ICC defined patients may be classified as a subgroup found within the broader category of CFS/ME [38]. Though broad criteria may be particularly useful for the identification of potential cases among small samples, it could inadvertently select those that do not have the illness [39]. The symptoms of chronic fatigue, post-exertional malaise, short-term memory and concentration problems reported in 1994 CDC defined CFS/ME cases are found to overlap with cases of depression [40]. The Canadian definition however, has been shown to effectively differentiate between those with CFS/ME and depression [17]. In another study, 96% of self-reporting cases of CFS/ME were found to comply with the 1994 CDC, compared to 77% of that complied with the Canadian definition [36]. These findings are consistent with the findings of this study, which is based on a similar method of recruitment and case ascertainment as, 91% of self-reporting cases fulfilled the 1994 CDC definition, 60% also fulfilled the Canadian definition, and 49% fulfilled the 1994 CDC, Canadian and ICC definitions.

An important consideration is a consistent method of applying criteria [41]. Reliance on self-reporting versus evaluation of cases by a physician can be a particular source of variability in reported cases of CFS/ME [42]. The symptom checklist used to verify the study criteria is limited to self-reporting of symptoms present during the past 30 days, and reporting of existing physical and psychiatric diagnoses. Self-reporting however, can be a useful tool for the initial evaluation of cases of research. Like the Canadian definition, the ICC definition was devised for clinical applications. Accordingly, the International Primer was published in 2012 [31], to aid clinicians in their evaluation of symptoms according to the ICC definition. The availability of this tool could contribute to the selection of homogenous patient sets in research settings and help exclude other causes of illness. The current primer however, does lack specifications on how to apply the definition in a research setting. This includes quantifiable measures on the severity and frequency of symptoms, in order to be considered significant in CFS/ME and its operationalization is discussed further by Johnston et al. [41].

As part of recruitment however, this study screened patients for reported medical history of chronic conditions such as heart disease, diabetes, and primary psychiatric disorder, as well as conducted standard blood tests as part of routine screening of disease. While all CFS/ME patients were found to have different results from controls in some parameters, no difference was found between the 1994 CDC and ICC patient groups. If the ICC definition identifies a subgroup with a less heterogeneous clinical presentation, important differences may be found in more specific biological markers than the ones examined in this study. While basic haematological abnormalities have not been previously reported in CFS/ME in relation to FBC, ESR, CRP and electrolytes, immunological abnormalities have been increasingly documented in cases of CFS/ME. Subsequently, salient differences have been detected in immunological parameters between 1994 CDC and ICC patient subgroups. In particular, significant differences in human neutraphil antigen [43].

The SF-36 survey that was used to evaluate the study groups is widely recognised as a valid and reliable tool for assessment of physical and social functioning in chronic illnesses [21], and has previously demonstrated impairment in 1994 CDC cases of CFS/ME [35,44,45]. It has also been used to contrast between patients fulfilling the 1994 CDC and Canadian definitions [36]. The comparison between 1994 CDC and ICC defined patients has been made in a US sample [38]. ICC defined patients reported significantly greater impairment in their physical functioning, bodily pain, physical role, as well as social functioning, which is consistent with the results from the Australian sample of this study. The WHO DAS 2.0 was further found to support the findings from the SF-36. Hence, there is evidence to suggest that inclusion of more symptoms in the ICC definition may select a significantly more impaired group.

The 1994 CDC definition for CFS/ME can represent an illness that ranges from mild impairment in daily activities, to severe cases where patients are bedridden and unable to care for themselves. For many chronic illnesses, the most severe cases often present themselves to primary or secondary care and mild cases often go unreported. The characteristics of CFS/ME can be quite the opposite, as the most debilitating cases can leave patients housebound or bedridden, and often overlooked by clinicians and researchers alike. The use of broad criteria with symptoms that overlap with other conditions can also make cardinal features of CFS/ME difficult to identify.

The current study suggests that the ICC definition may identify a more severe subgroup found within traditional CFS/ME and this may be consistent across samples in different geographic locations. The I994 CDC definition is limited to a nominal list of symptoms that may only capture the basic characteristics of the illness. As the ICC definition introduces a spectrum of symptoms affecting the neurological, immune and metabolic systems, it is expected that patients experiencing these further manifestations be more debilitated.

Limitations in this study include the application of the ICC definition as further research and consistency is required on the operationalization of the criteria that is not currently found in the primer. This study relied on whether patients had experienced any of the symptoms within the past 30 days. This time period is consistent with the time period measured in the SF-36 and WHO DAS 2.0. However, introducing thresholds for severity and frequency may provide further insight on the impact of this illness. It was also found that the majority of those that also fulfilled the ICC definition were female. To investigate whether females tended to report greater symptom severity than males in this subgroup, further comparison needs to be made in a larger sample size. A larger sample would also allow for further analysis of critical symptoms. This could contribute to more accurate and homogenous patient sets for further research on the aetiology and underlying pathomechanism of the illness.

The ICC definition has suggested that this subgroup should be referred to exclusively as Myalgic Encephalomyelitis (ME) patients. This proposal however, remains controversial as the term implies inflammation of the central nervous system that is not necessarily exhibited in all cases that fulfil the criteria. The term ME alone, may be viewed as pathogen-related initiation associated with onset as seen in bacterial, viral or parasitic infection and resultant inflammation of the nervous system. However use of the term in this context may result in misleading assignation of the syndrome directly and solely to an infectious agent. This has been seen previously during the XMRV expedition [46-51]. Alternatively the identification of this illness as due to immune dysfunction following infection or other initiating event represents a paradigm in closer fit with observable clinical signs and laboratory findings. Ongoing discoveries in immune dysfunction are likely to harmonise with more accurate case definitions over time.

## Conclusions

The preliminary findings of this study suggest that the ICC definition identifies a group of patients (generally females) that reported significantly greater disability, poorer social functioning, and ‘cognitive’ difficulties in comparison to the 1994 CDC definition. Though based on a small sample, these findings in an Australian sample are highly consistent with those previously reported in a cohort in the USA. This study included further comparison of standard blood tests but found no difference between the two patient groups. Further studies on the potential of the ICC definition to identify a unique and homogenous subgroup, and whether their reported physical state aligns with biological abnormalities will be of benefit for examining whether more specific biological markers are present in this illness.

## Competing interests

The authors declare that they have no competing interests.

## Authors’ contributions

SJ Contributed to the study design, data collection and analysis for this study. SJ, EB, DS, SM Contributed to the drafting and revisions of this manuscript. SH, TH, and EB Contributed to blood collection, and biological analysis for this study. All authors read and approved the final manuscript.
